# Pre-medical students’ perceptions of educational environment and their subjective happiness: a comparative study before and after the COVID-19 pandemic

**DOI:** 10.1186/s12909-021-03065-0

**Published:** 2021-12-15

**Authors:** Yanyan Lin, Ye Ji Kang, Hyo jeong Lee, Do-Hwan Kim

**Affiliations:** Department of Medical Education, Hanyang University College of Medicine, 222 Wangsimni-ro, Seongdong-gu, Seoul, 04763 Republic of Korea

**Keywords:** Dundee ready educational environment measure, Educational environment, Happiness, Professional identity, COVID-19

## Abstract

**Background:**

The COVID-19 pandemic necessarily changed pre-medical students’ educational environment into an online format—and students’ subjective happiness (SH) is highly impacted by their educational environment. This study investigates changes in pre-medical students’ perceptions of their educational environment and their SH before and after the pandemic, as well as explores the predictors related to their SH.

**Methods:**

The Korean version of the Dundee Ready Educational Environment Measure (DREEM) questionnaire and single-item measures of SH and professional identity (PI) were used. The t-test was employed to analyze the differences of the SH, PI, and DREEM subscales scores before and after the onset of COVID-19. Cohen’s d was used as effect size and correlations between SH and different subscales of DREEM were analyzed using Pearson’s correlation. The multiple regression analysis was performed to reveal associations between predictors and SH.

**Results:**

A total of 399 pre-medical students completed the survey both before and after the COVID-19 pandemic. The DREEM scores and all subscales scores significantly increased but each presents a different effect size. Students’ Perceptions of Learning (SPL: Cohen’s d = 0.97), Students’ Perceptions of Teaching (SPT: Cohen’s d = 1.13), and Students’ Perceptions of Atmosphere (SPA: Cohen’s d = 0.89) have large effect sizes. Students’ Academic Self-Perceptions (SASP: Cohen’s d = 0.66) have a medium effect size and Students’ Social Self-Perceptions (SSSP: Cohen’s d = 0.40) have a small effect size. In contrast, no significant change was noted in the SH and PI. Both PI and SSSP impacted SH before COVID-19, but after the pandemic, SH was impacted by SPL, SPA, and SSSP.

**Conclusions:**

Students’ overall perception of their educational environment was more positive after the onset of COVID-19, but their social self-perceptions improved the least. Additionally, SSSP is the only predictor of SH both before and after the pandemic. The findings of this study suggest that educational institutions must pay attention to students’ social relationships when trying to improve their educational environment. Furthermore, so as to increase students’ SH, development of both educational environment and PI is essential.

## Background

The COVID-19 pandemic has significantly impacted the educational environment of medical schools worldwide, and Korea is no exception [1]. For the pre-clerkship learning environment, the pandemic prohibited students from occupying lecture halls or small-group rooms, and the entire pre-clerkship curriculum, that included basic sciences and health system sciences, shifted online [2]. Clerkship rotations were postponed to combat the virus transmission, until social distancing was eased [3]. Thus, the pandemic affected both the pre-clinical and clinical learning environments. However, with the lockdown restrictions gradually easing, clinical rotations are now being rearranged [4–7]. Nevertheless, pre-medical students, as part of pre-clinical students, grappled with the online curriculum because of social distancing requirements [4].

Despite the sudden transition to online learning, medical students were highly accepting of the online format and were generally satisfied with the online course [4, 8–10]. Several studies reported students’ positive perception of online learning, and pre-clinical students, especially, were reported to appreciate online learning. It is well-established that students’ perceptions of the learning environment impact their professional identities [5, 9, 11]. However, despite a generally positive outlook toward the online format, many students also reported struggling with isolation and missing their interpersonal relations owing to the social distancing policy [12].

Previous studies have reported that social self-perception influences the subjective happiness (SH) of medical students [13]. Happiness is defined as “a global evaluation of life satisfaction,” and happiness is often referred to as subjective well-being [14]. Recent studies highlight the importance of medical students’ emotional well-being, considering its association with their academic performance and empathy [15, 16]. There was also a value proposition for a healthcare institution to increase social connectedness and enhance well-being as a basic human need [17]. As medical students’ SH is influenced by their perceptions of educational environment [13], it is necessary to investigate this factor to study the change in their SH level.

The Dundee Ready Educational Environment Measure (DREEM) is widely used for evaluating the medical educational environment, which includes learning, teaching, academic atmosphere, and social relationships among medical students [18–20]. Recently, a few researchers used the DREEM to study the relationship between the educational environment and students’ well-being. One study found that positive perceptions of the dentistry learning environment had a significant effect on students’ stress [21]; another found Student’s Social Self-perceptions—among the DREEM subscales—correlate significantly with SH [13]. However, few studies have examined this relationship in the context of COVID-19.

Numerous studies have focused on student burnout, depression, and anxiety during the COVID-19 [22, 23], but studies on students’ SH are scarce. Furthermore, the existing studies on medical students’ happiness and stress post-COVID-19 did not consider pre-pandemic data [22, 24–27]. Therefore, based on these studies, it is difficult to compare whether students’ perceptions have become more positive or negative post-COVID-19. To the best of our knowledge, few studies have investigated both students’ perceptions of educational environment and their happiness before and after the pandemic. This study was originally designed for another purpose before the onset of COVID-19, and thus, we had pre-pandemic data to compare with post-pandemic data. Consequently, we could examine the changes in medical students’ perceptions of educational environment and their SH because of COVID-19.

This study intends to discuss the following research questions in order to understand pre-medical students’ perception of educational environment and their SH. First, how have their perceptions of medical educational environment and SH changed after the COVID-19 pandemic. Second, what is the correlation between pre-medical students’ happiness and their perceptions of medical educational environment.

## Methods

### Study design and settings

This cross-sectional study was conducted at the Hanyang University College of Medicine in Korea. The institution’s curriculum is split into three phases: the initial 2 years comprise the pre-medical phase (PM1, PM2); the next 2 years cover the pre-clinical phase; and the final 2 years are called the clinical clerkship phase. The pre-medical courses are designed for cultivating an identity as a medical student (physician) and acquiring basic medical knowledge (humanities, basic sciences, medical terminology etc.). There was a minor curriculum revision at the beginning of the first semester of 2020, when, due to the COVID-19 pandemic, pre-medical courses were shifted online. In order to evaluate the curriculum revision, a questionnaire designed by the Curriculum Committee of the institution was distributed to the pre-medical students.

### Data collection

All pre-medical students were eligible to participate in this study. For the survey conducted in 2019, first year pre-medical (PM1) students (*n* = 114) and second year pre-medical (PM2) students (*n* = 100) participated. The paper-based survey was administrated to PM1 and PM2 students in their core courses (it was compulsory for all students to attend). Before collecting data, the purpose of the survey was explained orally. Students were informed that their participation was voluntary and unpaid, and that their answers would not influence their study results. For the 2020 survey, the same questionnaires and explanations were distributed electronically; because the curriculum changed to an online format after the pandemic, PM1 (*n* = 105) and PM2 students (*n* = 117) were asked to print and answer the questionnaires, and submit them to the researcher individually. The 2019 and 2020 surveys were both conducted in December, at the end of the semester, as they aimed to evaluate the curriculum. Most PM1 students in 2019 had become PM2 students in 2020.

In total, 403 students responded to the survey. We excluded incomplete questionnaires. However, if only one item from the DREEM measurement was omitted, these questionnaires were included after we replaced the item with the average value of the remaining items. Thirty-seven students were excluded, as they either did not return the questionnaire or returned incomplete ones. Finally, 399 questionnaires were analyzed (response rate = 91.5%).

### Measures

Participants were invited to complete two measures: 1. Korean version of the DREEM questionnaire; and 2. Single-item measures of happiness and professional identity (PI).

1. Korean version of DREEM

The DREEM was developed and verified by an international team to evaluate the educational environments of medical schools and other health training settings. Today, the DREEM is used in 20 countries worldwide and has been translated into eight languages. This study used the Korean version of the DREEM survey—translated by the Korean Society for Medical Education for a nationwide analysis of all medical schools in South Korea. Cronbach’s alpha—used to check the internal consistency of a subscale—of this version is comparable to the original DREEM and published studies of the survey’s translations in other languages [20, 28]. Furthermore, a validation study for a Korean version of the DREEM demonstrated that its five-factor structure was acceptable [29]. The Korean version of this survey has been widely used to measure the medical educational environment, including examining the relationship between students’ perception of the educational environment and their subjective happiness [13, 30, 31]. The survey, which includes 50 questions, employs a 5-point Likert scale (4 = strongly agree 0 = strongly disagree), divided into five subscales. Nine negative items (4,8,9,17,25,35,39,49, and 50) are scored in reverse order. The subscales and number of questions are as follows: Students’ Perception of Learning (SPL): 12; Students’ Perception of Teaching (SPT): 11; Students’ Academic Self-Perceptions (SASP): 8; Students’ Perceptions of Atmosphere (SPA): 12; and Students’ Social Self-Perceptions (SSSP): 7. As items NO.6 (SPT), NO.11 (SPA), and NO.18 (SPT) relate to the educational environment in the clinical setting, they were removed from this study. Regarding individual items, a mean score of 3.5 or greater regards educational environment as positive; a mean score between 2 and 3 signifies that it could be improved; and a mean score of 2 or less suggests problematic areas.

2. Single-item measures of happiness and professional identity.

We assessed the subject happiness using the item “To what extent do you think you are living a happy life?” in Korean. Response options ranged from “0 = not at all” to “10 = a great deal” on an 11-point scale. This single-item measure of happiness was used by a previous study to measure the SH of medical students of all phases in a medical school in Korea [11]. It was suggested in another study that the overall sense of well-being may be assessed quickly and efficiently through a brief measure of happiness [23]. Additionally, measuring happiness using a single item is reliable, valid, and viable [24]. In order to measure pre-medical students’ perceptions of the importance of PI (Professional identity) after they entered medical school, we used the following item: “Future Physician (Prospective Doctor) is one of the main aspects of my identity and I consider its importance is...?” The level of importance ranges from “1 = not at all” to “6 = a great deal.” In order to reduce the size and complexity of the questionnaire for participants and improve the response rate [32], we used this measurement. An analysis of previous studies revealed the lack of well-developed PI assessment methods for Korean pre-medical students. Furthermore, the single-item measure of other disciplines’ identity research showed evidence for validity and reliability [33]. The general information questionnaire was used to gather information about gender, age, grade, and GPA from respondents anonymously.

### Data analysis

After data pre-processing, 399 samples were identified as valid responses. Principally, the DREEM scores are reported as total scores of five subscales. In this study, we used the mean scores of items because of the deleted items (NO.6, NO.11, NO.18) as mentioned above. The data from the demographic questionnaire, the DREEM, SH, and PI were manually entered into SPSS version 26 (IBM Corp, USA) by one of the authors.

1. The t-test was used to analyze the differences in each of the SH, PI, and DREEM subscales score before and after COVID-19. As we had removed three items from the original DREEM questionnaire, we used the mean scores of items to compare the DREEM subscales scores difference. Cohen’s d was used as effect size, classifying effect sizes equal to.20,.50, and.80, respectively as small (negligible practical importance), medium (moderate practical importance), and large (crucial practical importance) effects, respectively [27].
2. Pearson’s r was calculated to examine whether there was an association between SH and DREEM subscales scores or PI, age, and GPA.
3. The multiple regression analysis was performed to assess the influence of DREEM subscales and PI on SH before and after COVID-19.

### Ethics statement

This study was reviewed and approved by the Institutional Review Board of the Hanyang University (approval No. HYU- 2020-04-002).

## Results

### Basic demographic characteristics

We received 399 valid questionnaires. Among the respondents, 175 students completed the questionnaire in Group 2019 and 224 students in Group 2020 (Table 1). The estimated response rate was 91.51% based on a total of 436 students. Participants were predominately male—among the 398 students who shared information about their gender, 304 were males (76.38%) and 94 were females (23.62%). The gender ratios in 2019 (74.14% males) and 2020 (77.88% males) were similar. The mean age was 20.03 (±1.25) years in Group 2019 and 20.28 (±1.92) years in Group 2020; the mean ages in the two groups showed no significant difference (*p* = 0.117). The students who provided their previous GPA in the two groups presented significant differences (*p* = 0.01) and the increased GPA may be because of the changed grading policy, which resulted in an increase in A and B grades after the curriculum shifted online.

**Table 1:** Descriptive statistics of survey respondents (*n* = 399)

Characteristic	2019	2020	Total
Grade			
PM1	103	111	214
PM2	72	113	185
Total	175	224	399
Gender			
Male	128	176	304
Female	46	48	94
Age (yr) Mean (SD)	20.03 ± 1.25	20.28 ± 1.92	20.17 ± 1.67
GPA Mean (SD)	4.34 ± 1.27	4.75 ± 1.22	4.57 ± 1.25

*PM* Pre-medical, *GPA* grade point average, *SD* Standard Deviation

GPA1: x< 2.0, GPA2: 2.0≦x<2.5, GPA3: 2.5≦x<3.0, GPA4: 3.0≦x<3.5, GPA5: 3.5≦x<4.0, GPA6: x>4.0

Six students disregarded age; the value of the omission was replaced with the average of age

One student disregarded gender

### Comparison of educational environment and subjective happiness before and after COVID-19

We used the t-test to compare the changes in students’ SH, PI, and perception of medical educational environment. Table 2 summarizes the statistically significant differences that were found. The DREEM scores (*p* < 0.001, Cohen’s d = 1.01) significantly increased after the COVID-19 epidemic and yielding a large effect size. All scores of subscales significantly increased but each yielded a different effect size. Students’ Perception of Learning (SPL) (*p* < 0.001, Cohen’s d = 0.97), Students’ Perception of Teaching (SPT) (*p* < 0.001, Cohen’s d = 1.13), and Students’ Perceptions of Atmosphere (SPA) (*p* < 0.001, Cohen’s d = 0.89) had large effect sizes. Students’ Academic Self-Perceptions (SASP) (*p* < 0.001, Cohen’s d = 0.66) had medium effect size and Students’ Social Self-Perceptions (SSSP) (*p* < 0.001, Cohen’s d = 0.40) had small effect size. In contrast, no significant change was noted in the SH (*p* = 0.257) and PI (*p* = 0.157) after COVID-19.

**Table 2:** Subjective happiness, professional identity, and DREEM subscales scores from 2019 and 2020

		N	M	SD	F	P	t(df)	p	Cohen’s d
SH	2019	171	6.71	1.82	0.11	0.736	−1.13(394.00)	0.257	0.12
	2020	225	6.92	1.77					
PI	2019	175	3.69	1.82	3.70	0.005	−1.42(389.00)	0.157	0.15
	2020	216	3.94	1.64					
DREEM	2019	172	2.09	0.41	3.64	0.057	−10.07(396.00)	0.000	1.01
	2020	226	2.55	0.49					
SPL	2019	172	1.86	0.55	2.08	0.150	−9.52(396.00)	0.000	0.97
	2020	226	2.42	0.60					
SPT	2019	172	2.24	0.54	0.29	0.591	−11.17(396.00)	0.000	1.13
	2020	226	2.87	0.57					
SASP	2019	172	2.24	0.54	0.51	0.475	−6.40(396.00)	0.000	0.66
	2020	226	2.61	0.58					
SPA	2019	172	2.03	0.46	8.16	0.005	−9.03(393.62)	0.000	0.89
	2020	226	2.49	0.56					
SSSP	2019	172	2.21	0.41	12.94	0.000	−4.15(395.51)	0.000	0.40
	2020	226	2.41	0.56					

*SH* Subjective Happiness, *PI* Professional Identity, *GPA* grade point average, *DREEM* Dundee Ready Educational Environment Measure, *SPL* Students’ Perception of Learning, *SPT* Students’ Perception of Teaching, *SASP* Students’ Academic Self-Perceptions, *SPA* Students’ Perceptions of Atmosphere, *SSSP* Students’ Social Self-Perceptions

### Correlation between subjective happiness and educational environment and professional identity

A correlate analysis (Table 3) confirmed a significant positive relationship between all subscales of DREEM and SH (SPL: *r* = 0.288; SPT: *r* = 0.218; SASP: *r* = 0.352; SPA: *r* = 0.356; SSSP: *r* = 0.433, *p* < 0.001). Results also confirmed a significant relationship between PI and SH (*r* = 0.223, *p* < 0.001). However, there was no significant association with the age and GPA of the students in statistics.

**Table 3:** Correlation between several possible determinants of students’ subjective happiness

	SH	PI	Age	GPA	SPL	SPT	SASP	SPA	SSSP
SH	1								
PI	.223**	1							
Age	−0.012	−0.029	1						
GPA	0.03	0.092	−.233**	1					
SPL	.288**	.217**	0.08	.153**	1				
SPT	.218**	.114*	0.001	.124*	.705**	1			
SASP	.352**	.246**	0.071	.228**	.723**	.482**	1		
SPA	.356**	.203**	−0.003	.160**	.820**	.674**	.661**	1	
SSSP	.433**	.192**	0.018	0.016	.623**	.387**	.602**	.643**	1

**P < 0.01, *P < 0.05 using a two-tailed test

*SH* Subjective Happiness, *PI* Professional Identity, *GPA* grade point average, *SPL* Students’ Perception of Learning, *SPT* Students’ Perception of Teaching, *SASP* Students’ Academic Self-Perceptions, *SPA* Students’ Perceptions of Atmosphere, *SSSP* Students’ Social Self-Perceptions

### Predictors of subjective happiness before and after COVID-19

The multiple linear regression analysis was conducted to further examine the associations between predictors and SH (Table 4). Gender, Grade, GPA, Age, and PI were entered into the analysis. The results demonstrated that PI (β = 0.251, *p* = 0.001) and SSSP (β = 1.416, *p* = 0.001) were significantly associated with SH in 2019. The variables explained 24.8% of students’ SH. In 2020, SPL (β = − 0.998, *p* = 0.007), SPA (β = 1.154, *p* = 0.005), and SSSP (β = 1.063, *p* = 0.001) were significantly associated with SH. These variables were determinants of 21.6% of the variance of students’ SH.

**Table 4:** Multiple regression analysis

	Dependent Variable	Independent Variables	Unstandardized Coefficients		Standardized Coefficients	t	*p*-value	Adj R2
Group	SH		B	Standard error	β			
2019			2.893	2.75		1.052	0.294	0.248(6.473)
		Gender	0.268	0.299	0.065	0.895	0.372	
		Grade	−0.119	0.29	−0.032	−0.411	0.682	
		GPA	−0.086	0.114	−0.059	− 0.753	0.453	
		Age	−0.078	0.126	−0.049	−0.623	0.534	
		PI	0.251	0.076	0.241	3.313	0.001	
		SPL	−0.253	0.436	−0.075	−0.58	0.563	
		SPT	0.164	0.327	0.048	0.5	0.618	
		SASP	0.547	0.323	0.161	1.693	0.092	
		SPA	0.18	0.407	0.046	0.443	0.659	
		SSSP	1.416	0.411	0.318	3.446	0.001	
2020			2.913	1.607		1.812	0.071	0.216(6.728)
		Gender	0.103	0.285	0.023	0.362	0.718	
		Grade	−0.113	0.267	−0.031	−0.423	0.673	
		GPA	−0.064	0.106	−0.042	− 0.603	0.547	
		Age	0.007	0.062	0.007	0.109	0.913	
		PI	−0.002	0.071	−0.002	− 0.025	0.98	
		SPL	−0.998	0.368	−0.334	−2.709	0.007	
		SPT	0.137	0.276	0.044	0.498	0.619	
		SASP	0.33	0.319	0.105	1.034	0.302	
		SPA	1.154	0.402	0.363	2.87	0.005	
		SSSP	1.063	0.302	0.335	3.524	0.001	

*SH* Subjective Happiness, *PI* Professional Identity, *GPA* grade point average, *SPL* Students’ Perception of Learning, *SPT* Students’ Perception of Teaching, *SASP* Students’ Academic Self-Perceptions, *SPA* Students’ Perceptions of Atmosphere, *SSSP* Students’ Social Self-Perceptions

## Discussion

In this study, we use the DREEM to evaluate pre-medical students’ perceptions of medical educational environment and their SH during the COVID-19 epidemic and explore the changes related to the predictors of students’ SH. We found that DREEM scores and its five subscales scores all increased after the onset of COVID-19, but not in SH and PI. The predictors associated with student SH were the PI and the social self-perception (SSSP). However, during the pandemic, the perception of the atmosphere (SPA) became a new predictor associated with SH; the PI was no longer the predictor.

### DREEM scores increased after the onset of COVID-19

Contrary to expectations, this study found that the overall DREEM scores and subscales scores all increased. These findings are consistent with those of previous studies, in that most of the pre-clinical students had a positive perception of future online learning after the sudden transition during the pandemic [9]. The learning environments can be broadly defined as physical, virtual, and sociocultural spaces in which learning takes place [34]. Considering this definition and the advantages of online learning during the pandemic, we can explain the improved perceived learning environment. Some studies have shown that students perceive the ability to make their choices of space and time to study as a benefit of online learning, which can improve the physical learning environment [10, 35]. Students enjoyed the self-paced learning of video lectures [36] and online accessible educational material [35], −both examples of virtual categories [34]. The online environment provided a fair chance to facilitate positive interaction with the professor [10], that may also promote the perception of the sociocultural spaces. Thus, the online learning implemented after the pandemic may have contributed to the increase in the overall DREEM scores.

Furthermore, all subscales of DREEM scores improved after the onset of COVID-19; however, we found that the effect sizes of subscales were different. The SPT (Cohen’s d = 1.13) improved the most and the SSSP (Cohen’s d = 0.40) improved the least, based on the effect size. As discussed above, positive opinions about online learning’s comfortable environment [10] may have played a vital role in bringing about the greatest increase in scores for the SPT. Answers about one’s attitudes in surveys can be affected by the aforementioned items’ answers, so improvements in SSSP may be based on the improvements in other items in this questionnaire. Additionally, mixed items about teaching and learning in the DREEM questionnaire, which contributed to the overall increased DREEM scores may have a context effect on the increased score in the SSSP [37].

### Subjective happiness, professional identity, and DREEM scores

No differences were found in students’ SH and PI after COVID-19; however, non-significant changes in SH match were observed in another study in Germany [38]. These results were in contrast to earlier findings of negative impact on students’ mental health, such as depression and anxiety, due to COVID-19 [22, 23]. What we know about SH is related to many factors: relationships, physical health, mental health, and financial success [39]. This suggests that many factors can affect students’ SH. Social connectedness and academic stress may have negative impacts on students, whereas spending more time with family, exercise, and sleep may have positive impacts on them [22]. It is, therefore, likely that SH is balanced by such causes. Professional identity formation is a dynamic and non-linear dimension that exists at every level of medical education, and is impacted by socialization [40]. We found that PI did not change after the pandemic, which can be explained as follows. Isolation from peers and teachers may have a negative influence on socialization but a program like “1-hour national Twitter discussion by the Becoming a Doctor (BAD) team” through social media to support students during the pandemic may have a positive influence on socialization [41]. Additionally, a previous study found that medical students’ preferred activities to counter the adverse impacts of the pandemic include social media use, which is known to have potential benefits for professional development [22, 42, 43]. Such instances of balanced socialization may have balanced the students’ PI during the COVID-19 pandemic.

In this study, the five subscales of DREEM all correlated with students’ SH. These results are different from earlier findings that show that only SASP (academic self-perceptions), SPA (perceptions of atmosphere) and SSSP (social self-perception) are correlated to SH (subjective happiness) in pre-medical students. However, the finding that GPA did not contribute to SH concurred with previous research [13]. One implication of this is that changes caused by COVID-19 changed the predictors for students’ SH.

We further analyzed the predictors for SH using the multiple regression analysis by controlling for other variables before and after COVID-19. The results indicate that before the pandemic, professional identity (PI) and SSSP were associated with SH, but afterwards, SPL (perception of learning), SPA, and SSSP were related to SH. It is interesting to note that SSSP was a common predictor of SH, regardless of the pandemic. This finding confirms the positive influence of SSSP on SH and is consistent with the idea that socialization is important for SH [13]. Another finding of this study showed that PI is a positive predictor of SH. Previous studies have demonstrated that the greater the importance of identity, the more one feels important to others, which enhances the purpose of life. A purposeful life makes people happier, and, therefore, higher PI may increase one’s happiness [44, 45].

As mentioned above, online learning has the advantages of flexibility and physical comfort, which may be the reason why SPA has a positive impact on SH [10]. However, one unanticipated finding was that SPL negatively influenced SH after the onset of COVID-19, which may be because of the following reason. Pre-medical students in Korea are accepted to medical schools through a tough competition, and, therefore, consider themselves as the best students of their respective high schools; they also have a tendency to be perfectionists [46, 47]. Consequently, they may feel a lot of pressure to obtain high academic scores in medical school. For example, they may watch online recorded lectures repeatedly to obtain a perfect score, which may engender high academic stress, and higher academic stress could be attributed to low happiness [10, 48].

### Limitations

This study’s design as a single-center analysis limits generalizability to other settings. In this study, we used single-item measures of happiness and PI. This decision is based on previous studies that measured medical student’s SH [13, 24, 49] and medical students’ and science, technology, engineering, and mathematics (STEM) students’ PI [33, 50], and showed that SH and PI can be validly and reliably evaluated via single-item measurement. Additionally, in order to increase the response rate, a single-item measure for SH and PI is used [32].

### Future study and implications

The findings of this study have some important implications for practice. First, because SSSP scores improved the least (based on the effect size) after the onset of COVID-19 compared to the other subscales, educators must focus on improving students’ ﻿social self-perceptions when face-to-face activities are impossible. As shortage of time for relaxation or relationships is a common challenge for medical students [51], it is imperative to improve their social self-perception. Second, the results show a positive association between SH and PI. There is a considerable degree of instability about happiness, which can depend on contextual circumstances [52]. Moreover, external components, such as environmental factors and income, can also affect happiness [16]. Thus, when the external environment changes adversely—as it did in the case of COVID-19—we can educate students to invoke their PI to improve their SH. It is also important that medical students actively participate in social activities and form healthy interpersonal relationships—even if only through an online medium—to enhance their happiness. Medical schools and institutions should conduct regular psychological surveys, pay attention to the psychological changes in students, and improve their learning environment. Our results suggest that there is a great need to explore medical students’ SH and PI in relation to their educational environment. The goal of researching medical students’ happiness is to equip them for the future healthcare system and improve patient care. Future research should investigate variables that correlate with SH and PI, to improve medical students’ SH and PI.

## Conclusions

The main goal of the current study was to determine the changes in pre-medical students’ perception of the educational environment and their SH after COVID-19 as well as to understand how pre-medical students’ perceptions of different aspects of the educational environment and PI affect their SH. The first finding of this study is that, according to the students we surveyed, pre-medical students’ perceptions of the educational environment improved following COVID-19. However, no significant change was acclaimed in the students’ SH and PI. The second major finding was that all subscales of DREEM and the PI correlated to SH. The third finding was that PI and SSSP emerged as reliable predictors of SH before COVID-19, whereas SPL, SPA, and SSSP after the epidemic. The findings of this study suggest that institutions need to pay attention to student’s social life when improving the educational environment. Furthermore, to increase students’ SH, promotion of educational environment as well as the development of PI will be needed.

## Data Availability

The datasets of this article are available from the corresponding author on reasonable request.

## Abbreviations

SH: Subjective Happiness
PI: Professional Identity
DREEM: Dundee Ready Educational Environment Measure
SPL: Students’ Perception of Learning
SPT: Students’ Perception of Teaching
SASP: Students’ Academic Self-Perceptions
SPA: Students’ Perceptions of Atmosphere
SSSP: Students’ Social Self-Perceptions
GPA: Grade point average
